# Spinal decompression improves walking capacity in patients with lumbar spinal stenosis

**DOI:** 10.1016/j.bas.2025.104268

**Published:** 2025-04-28

**Authors:** Mikkel Ø. Andersen, Leah Y. Carreon, Stefan Hummel, Elisabeth C. Smith, Andreas K. Andresen

**Affiliations:** a Center for Spine Surgery and Research, Lillebaelt Hospital, Kolding, Denmark; b Institute of Regional Health Research, University of Southern Denmark, Campusvej 55, DK-5230, Odense M, Denmark

**Keywords:** Lumbar spinal stenosis, Neurogenic claudication, Spinal decompression, Walking speed, Walking distance

## Abstract

**Introduction:**

Lumbar spinal stenosis (LSS) is a degenerative condition causing back and leg pain, limiting walking due to neurogenic claudication. It affects 9–11 % of the population, rising to 47 % in those over 60, with cases expected to increase as the population ages. Non-surgical treatments are considered first-line options, although their effectiveness remains uncertain. Decompression surgery is still commonly performed for severe cases, even though a review comparing conservative treatments with surgical procedures, including spinal decompression, found no clear superiority of surgery. In Denmark, LSS accounts for 35 % of spinal surgeries in adults.

**Research question:**

Does spinal decompression improve walking distance and gait speed in patients suffering from LSS?

**Methods:**

Consecutive patients scheduled for decompression due to spinal stenosis enrolled at a regional spine centre. Timed walking distance (maximum of 1000m) was performed at baseline and at 3-, and 12 months post-op.

**Results:**

One hundred and one patients were included in the study, mean age was 70.7 years, 77 % were female, with 90 % having had symptoms for more than 6 months prior to surgery.

Walking distance (123.9m–791.1m, p < .001) and speed (0.91 m/s to 1.17 m/s, p < .001) improved at one year after surgery.

**Discussion and conclusion:**

Patients with severe walking impairment caused by spinal stenosis experienced substantial improvement of both walking speed and walking distance at all follow-up time points after undergoing spinal decompression.

## Highlights

• Spinal decompression improved overall walking distance and speed.
• Non-ambulatory patients regained significant walking ability.
• Walking speed improvement may add five years to life expectancy.

## 1 Introduction

Lumbar spinal stenosis (LSS) is a degenerative condition predominantly affecting elderly individuals, characterized by the narrowing of the spinal canal or neural foramen, leading to compression of neural and vascular structures. This condition typically manifests as low back pain as well as pain in the buttocks or lower extremities, accompanied by reduced walking distance due to neurogenic claudication, which worsens with prolonged standing or walking. The prevalence of LSS remains somewhat uncertain but is estimated to affect 9–11 % of the general population and up to 47 % of individuals over the age of 60 (Jensen et al., 2020). With the aging population, the incidence of LSS is expected to rise significantly.

Patients with LSS can present with mild symptoms, which can be treated with and be responsive to non-operative care. Non-operative care can include physiotherapy, medication, and lifestyle modifications, as recommended and mandatory in Denmark as first-line interventions for LSS. Surgical decompression is often the preferred treatment for severe cases of LSS or for patients who do not improve after extensive non-operative care. However, its clinical outcomes remain uncertain according to a 2016 Cochrane review (Zaina et al., 2016) based on studies comparing conservative treatments with surgical procedures.

In Denmark, LSS is the most frequent reason for spinal surgery in individuals over 65, accounting for 35 % of all spinal surgeries annually equivalent of 2000–2500 surgeries for LSS each year (DaneSpine, 2023). As the choice of treatment depends on patient preference, with non-operative care being recommended for mild to moderate disease and operative treatment offered only for severe disease or persistent symptoms, a true head-to-head comparison of non-operative treatment to operative treatment for LSS is not possible. That is, by the time the patient is a candidate for surgery, they are past equipoise, where the patient can be treated with either non-operative or operative care. In addition a true randomized trial cannot be performed as blinding non-operative to operative treatment is not possible.

Numerous studies have examined the impact of decreased walking ability and speed on morbidity and mortality. Several of these studies have demonstrated a link between gait speed and survival among older adults (Studenski et al., 2003; Markides et al., 2001; Woo et al., 1999; Guralnik et al., 1994). It has been noted that older individuals with a gait speed below 1 m/s are at a higher risk of experiencing adverse health outcomes, underscoring the importance of maintaining patients' walking ability and speed. The aim of this study was to investigate whether spinal decompression improves walking capacity and gait speed in patients with LSS. Given the importance of mobility in maintaining quality of life, answering this question is crucial for guiding treatment decisions in this growing patient population.

## 2 Material and methods

The study was a single center, prospective cohort study of patients undergoing lumbar decompression for LSS. Approval for the study was obtained from the Regional Committees on Health Research Ethics.

A consecutive series of patients who were referred to a regional spine center from the primary health care sector were recruited. Criterion for referral to the spine center was persistent symptoms after at least 3 months of non-operative treatment. All patients had Magnetic Resonance Imaging (MRI) performed to confirm the diagnosis of LSS prior to the consultation. A spine surgeon who had been in practice for at least 5 years examined patients. When the visit to the clinic was scheduled, patients underwent standing lumbar x-rays to rule out significant deformity of the spine.

Inclusion criteria were patients aged 60 and above, significant spinal stenosis at one or two levels from L1 to S1 verified on MRI, neurologic claudication with a Konno score of >6 (Konno et al., 2007) and completion of a minimum of 3 month of unspecified non-operative treatment in primary sector supervised by either a physiotherapist or chiropractor without improvement of symptoms.

### 2.1 Treatment

All patients underwent an open, midline-sparing decompression. In a prone position the skin level for decompression was marked under fluoroscopic guidance. The musculature corresponding to the relevant levels was dissected out to the facet joints, followed by a hemilaminectomy. Depending on the surgeons' preference, the procedures were performed with the assistance of either a microscope or loupes. Upon discharge, each patient received thorough guidance from a physiotherapist affiliated with the surgical department, focusing on initiating exercises and providing training instructions. At the three-month follow-up, the primary surgeon examined the patients, after which they began rehabilitation within the municipality.

### 2.2 Outcome measures

Demographic data regarding age, gender, height, weight, and comorbidities were collected from questionnaires and electronic patient records. Patient reported outcomes on back- and leg pain were measured on a 0–100 visual analogue scale (VAS) (Scott and Huskisson, 1976), where higher scores reflect more severe pain. The VAS pain was self-reported and measured prior to surgery, at 3 months and 12 months postoperative.

### 2.3 Walking test

The walking test was performed on a measured 25-m indoor track with a hard surface. Patients were instructed to wear comfortable shoes and walk around a cone positioned at each end of the track. Further, they were instructed to walk at their own preferred pace and to stop if they experienced symptoms that made them feel the need to rest or sit down. Before the test, each patient rested for at least 2 min while seated in a comfortable chair. The use of a cane or walker was permitted and recorded if needed. A physiotherapist supervised the test, and walking time was recorded to determine the average walking speed. The walking test was conducted preoperatively, and at 3 and 12 months postoperatively. Patients were asked whether they stopped walking due to leg pain, back pain, or a combination of both. The test was capped at a maximum distance of 1000 m.

### 2.4 Statistics

All continuous variables were tested for normality. Pre and post-operative variables were compared using independent paired *t*-test for normally distributed continuous variables, presented as means and 95 % confidence intervals, Wilcoxon rank was used for non-normally distributed continuous variables and Fisher's exact test for categorical data, presented as numbers and percentages. Statistical analyses were performed in STATA 18.0 (StataCorpLLC, College Station, TX) with a p-value of <0.05 considered statistically significant.

## 3 Results

The study included 101 patients with an average age of 70.7 years. Of these, 77 were female, 79 underwent single-level decompression, and two-thirds had experienced symptoms for over a year before undergoing surgery (Table 1).

**Table 1:** Baseline characteristics.

**Age, years, Mean (95 % Confidence Interval)**	70.7 (69.4; 72.0)
**Females, N (%)**	77 (76.2 %)
**Single-level decompression, N (%)**	79 (78.2 %)
**BMI∗, kg/m^2^, mean (95 % Confidence Interval)**	27.1 (26.3; 27.9)
**Hypertension, N (%)**	54 (53.5 %)
**Diabetes Mellitus, N (%)**	13 (12.9 %)
**Duration of symptoms, N (%)**	
3–6 months	11 (10.9 %)
>6–12 months	23 (22.8 %)
>12 months	67 (66.3 %)

Ninety-eight patients (97 %) responded to the pain questions and 94 (94 %) were available for the walking test at the 12-month follow-up. Prior to surgery, 89 % of patients had to stop walking due to leg pain or a combination of leg and back pain, 9 % stopped because of back pain alone, and 2 % did not stop walking at all. By the 12-month mark, 75 % could walk 1000 m without stopping, 21.3 % stopped due to leg or leg-and-back pain, and 4.3 % due to isolated back pain.

Preoperatively, seven patients (6.9 %) reported severe leg pain that completely prevented walking and were excluded from the walking speed analysis. At the 3-month follow-up, all seven were able to walk at least 300 m, with four reaching the 1000-m truncation limit. By 12 months, all but one patient reached the 1000-m truncation limit during the walking test.

Walking capacity showed significant improvement at 12 months post-surgery, with an average increase in walking distance of 660.2 m (CI 583.9; 736.9, p < .001) and a corresponding mean improvement in walking speed of 0.24 m/s (CI 0.18; 0.30, p < .001) (Table 2). The symptom duration significantly influenced the preoperative walking distance (p < .001); however, it had no effect on either the postoperative walking distance (p < .175) or the improvement in walking distance (p < .875).

**Table 2:** Summary of results.

	Preoperative	3 months Post-operative	12 months Post-operative	p-value
**Walking distance, m, Mean, (95 % Confidence Interval)**	123.9 (86.5; 161.2)	669.7 (593.5; 756.9)	791.1 (722.6; 859.7)	<0.001
**Walking speed, m/s, Mean, (95 % Confidence Interval)**	0.91 (0.86; 0.97)	1.08 (1.02; 1.15)	1.17 (1.12; 1.22)	<0.001
**Reason to stop walking, N (%)**				<0.001
Did not stop	2 (2 %)	57 (60 %)	70 (75 %)	
Leg pain	52 (51 %)	21 (22 %)	13 (14 %)	
Back pain	9 (9 %)	7 (7 %)	4 (4 %)	
Back and Leg pain	38 (38 %)	10 (11 %)	7 (8 %)	
**Oswestry Disability Index, mean (SD**)	39.4 (14.1)	19.8 (14.9)	18.1 (15.8)	<0.001
**EuroQol-5D, mean (SD)**	0.54 (0.20)	0.77 (0.15)	0.81 (0.17)	<0.001
**VAS– Leg pain, mean (SD)**	66.7 (18.9)	21.0 (23.8)	23.7 (26.5)	<0.001
**VAS- Back pain, mean (SD)**	55.6 (25.1)	20.7 (18.7)	22.4 (24.1)	<0.001

The mean preoperative VAS score for leg pain was 66.7 (SD 18.9), which improved to 21.0 (SD 23.8) at 3 months and remained at 23.7 (SD 26.5) at 12 months. For back pain, the mean VAS score before surgery was 56.7 (SD 25.1), showing significant improvement to 20.7 (SD 18.7) at the 3-month follow-up, and remaining stable at 22.4 (SD 24.1) by 12 months. Both the ODI and EQ-5D scores also showed significant improvement at both the 3- and 12-month postoperative follow-ups (Table 2).

## 4 Discussion

In this prospective cohort study of 101 patients with lumbar spinal stenosis, we found that objective measures for walking distance and walking speed were significantly improved after spinal decompression surgery. These outcomes align with prior published results (Smuck et al., 2018; Sakai et al., 2020), which also found notable improvements in walking speed following spinal decompression for LSS. Further, the present study showed a marked decrease in the number of patients who had to stop walking due to either leg or back pain, from 98 % preoperatively to less than 1/3 of the patients at the 12-month follow-up. Interestingly the main improvement occurred within the first 3 months after the surgery and prior to the supervised rehabilitation.

Walking speed, as an objective measure of physical function, is particularly interesting in patients with spinal stenosis. A meta-analysis involving 34,485 participants highlighted a strong association between walking speed and survival in the elderly regardless sex, body mass index, smoking status, systolic blood pressure or prior hospitalizations with faster walking speeds correlating with increased life expectancy (Studenski et al., 2011). In our cohort of 71 year olds, the observed improvement in walking speed could be equivalent to an increased life expectancy of approximately five years, underscoring the broader health benefits of restoring mobility through surgical intervention (Studenski et al., 2011).

The strength of this study is that walking speed was measured over the entire distance of 1000 m covered by this cohort of patients with spinal stenosis, providing a more accurate representation of everyday activity, where fatigue occurs during walking. This approach differs from previous studies, which assessed walking speed over only a 10-m distance or included significantly fewer patients.

The study has certain limitations, as it was a prospective cohort study rather than a randomized trial. It is unknown if walking distance and speed in these patients would have improved without surgery. But, the majority of patients included had experienced symptoms for over 12 months and had undergone at least 3 months of unsuccessful conservative treatment before choosing surgical intervention. This is significant because these patients are no longer candidates for continued non-operative treatment and it is well-established that prolonged symptom duration is associated with diminished patient-reported outcomes.

## 5 Conclusion

This study supports the role of spinal decompression surgery in significantly improving walking capacity and speed in patients with LSS. Given the aging global population and the expected rise in LSS prevalence, these findings are crucial in guiding treatment strategies and improving the quality of life for this patient population. Further studies with longer follow-up periods are warranted to explore the long-term benefits and risks of surgical intervention for LSS.

## Funding

This research did not receive any specific grant from funding agencies in the public, commercial, or not-for-profit sectors.

## Declaration of competing interest

The authors declare that they have no known competing financial interests or personal relationships that could have appeared to influence the work reported in this paper.
